# Metabolic Acidosis Is Associated With an Accelerated Decline of Allograft Function in Pediatric Kidney Transplantation

**DOI:** 10.1016/j.ekir.2024.04.007

**Published:** 2024-04-08

**Authors:** Agnieszka Prytula, Rukshana Shroff, Ineke van Gremberghe, Kai Krupka, Justine Bacchetta, Elisa Benetti, Ryszard Grenda, Isabella Guzzo, Nele Kanzelmeyer, Bahar Büyükkaragöz, Birgitta Kranz, Hülya Nalçacıoğlu, Jun Oh, Lars Pape, Mohan Shenoy, Anne-Laure Sellier-Leclerc, Burkhard Tönshoff, European Society for Paediatric Nephrology Transplantation and CKD-MBD working groups and the Cooperative European Paediatric Renal Transplant Initiative Research Network, Gema Ariceta, Atif Awan, Sevcan Bakkaloğlu, Marjolein Bonthuis, Charlotte Bootsma Robroeks, Antonia Bouts, Martin Christian, Marlies Cornelissen, Ali Duzova, Nasrin Esfandiar, Luciana Ghio, Ryszard Grenda, Isabella Guzzo, Maria Herrero Goni, Julien Hogan, Nattaphorn Hongsawong, Nele Kanzelmeyer, Aysun Karabay Bayazit, Gülşah Kaya Aksoy, Noel Knops, Linda Koster Kamphuis, Daniella Levy Erez, Victor Lopez-Baez, Alvaro Madrid, Stephen Marks, Anette Melk, Luisa Murer, Lars Pape, Licia Peruzzi, Edita Petrosyan, Evgenia Preka, Nikoleta Printza, Andreea Liana Rachisan, Ann Raes, Mohan Shenoy, Oguz Soylemezoglu, Luca Dello Strologo, Ana Teixeira, Rezan Topaloglu, Markus Weitz, Jakub Zieg, Galia Zlatanova, Christian Patry, Jerome Harambat, Ayşe Ağbaş, Varvara Askiti, Marina Avramescu, Justine Bacchetta, Sevcan Bakkaloglu, Marjolein Bontuis, Caroline Booth, Laurene Dehoux, Giacomo Dizazzo, Dorota Drozdz, Ismail Dursun, Michaela Gessner, Jaap Groothoff, Giuliana Guido, Isabella Guzzo, Aysun Karabay Bayazit, Guenter Klaus, Linda Koster-Kamphuis, Alexander Lalayiannis, Maren Leifheit-Nestler, Sinha Manish, Chiara Matteucci, Jun Oh, Ozan Ozkaya, Edita Petrosyan, Christine Pietrement, Agnieszka Prytula, George Reusz, Franz Schaefer, Claus Peter Schmitt, Anne Schön, Fatma Lale Sever, Stella Stabouli, Serra Sürmeli Döven, Camilla Tondel, Enrico Verrina, Enrico Vidal, Dean Wallace, Zainab Arslan, M. Bald, H. Fehrenbach, D. Haffner, M. Hansen, C. Hempel, U. John, G. Klaus, J. König, B. Lange-Sperandio, D. Müller, J. Oh, L. Pape, M. Pohl, K. Sauerstein, G. Schalk, H. Staude, P. Strotmann, L.T. Weber, M. Weitz, L. Berta, K. Heindl-Rusai

**Affiliations:** 1 Department of Pediatric Nephrology and Rheumatology, Ghent University Hospital, Belgium; 2 Renal Unit, UCL Great Ormond Street Hospital, London, UK; 3 Biostatistics Unit, Faculty of Medicine and Health Sciences, Ghent University, Belgium; 4 Department of Pediatrics I, University Children’s Hospital Heidelberg, Germany; 5 Department of Pediatric Nephrology and Rheumatology, CHU de Lyon, Bron, France; 6 Pediatric Nephrology, Dialysis and Transplant Unit, Laboratory of Immunopathology and Molecular Biology of the Kidney, Department of Women’s and Children’s Health, Padua University Hospital, Padua, Italy; 7 Department of Nephrology, Kidney Transplantation and Arterial Hypertension, Children’s Memorial Health Institute, Warsaw, Poland; 8 Division of Nephrology, Dialysis and Renal Transplant Unit, Bambino Gesù Children’s Hospital IRCCS, Rome, Italy; 9 Department of Pediatric Kidney, Liver and Metabolic Diseases, Hannover Medical School, Hannover, Germany; 10 Department of Pedaitric Nephrology, Gazi University, Ankara, Turkey; 11 Pediatric Nephrology, University Children‘s Hospital, Münster, Germany; 12 Pediatric Nephrology Department, Ondokuz Mayis University, Samsun, Turkey; 13 Department of Pediatric Nephrology and Transplantation, University Children’s Hospital, University Medical Center Hamburg/Eppendorf, Hamburg, Germany; 14 Department of Pediatrics II, University Hospital of Essen, Essen, Germany; 15 Royal Manchester Children’s Hospital, Manchester, UK

**Keywords:** acidosis, pediatric, transplantation

## Abstract

**Introduction:**

We investigated the relationship between metabolic acidosis over time and allograft outcome in pediatric kidney transplantation (KTx).

**Methods:**

This registry study collected data up to 10 years posttransplant. Survival analysis for a composite end point of graft loss or estimated glomerular filtration rate (eGFR) ≤ 30 ml/min per 1.73 m^2^ or ≥50% decline from eGFR at month 3 posttransplant was performed. The association of serum bicarbonate concentration (HCO_3_^−^) < 22 mmol/l (metabolic acidosis) and HCO_3_^−^ < 18 mmol/l (severe metabolic acidosis) with allograft outcome was investigated using stratified Cox models and marginal structural models. Secondary analyses included the identification of risk factors for metabolic acidosis and the relationship between alkali supplementation and allograft outcome.

**Results:**

We report on 1911 patients, of whom 347 reached the composite end point. The prevalence of metabolic acidosis over time ranged from 20.4% to 38.9%. In the adjusted Cox models, metabolic acidosis (hazard ratio [HR], 2.00; 95% confidence interval [CI], 1.54–2.60) and severe metabolic acidosis (HR, 2.49; 95% CI, 1.56–3.99) were associated with allograft dysfunction. Marginal structural models showed similar results (HR, 1.75; 95% CI, 1.32–2.31 and HR, 2.09; 95% CI, 1.23–3.55, respectively). Older age was associated with a lower risk of metabolic acidosis (odds ratio [OR] 0.93/yr older; 95% CI, 0.91–0.96) and severe metabolic acidosis (OR, 0.89; 95% CI, 0.84–0.95). Patients with uncontrolled metabolic acidosis had the worst outcome compared to those without metabolic acidosis and without alkali (HR, 3.70; 95% CI, 2.54–5.40)

**Conclusion:**

The degree of metabolic acidosis is associated with allograft dysfunction.

## Graphical abstract

**Figure:**
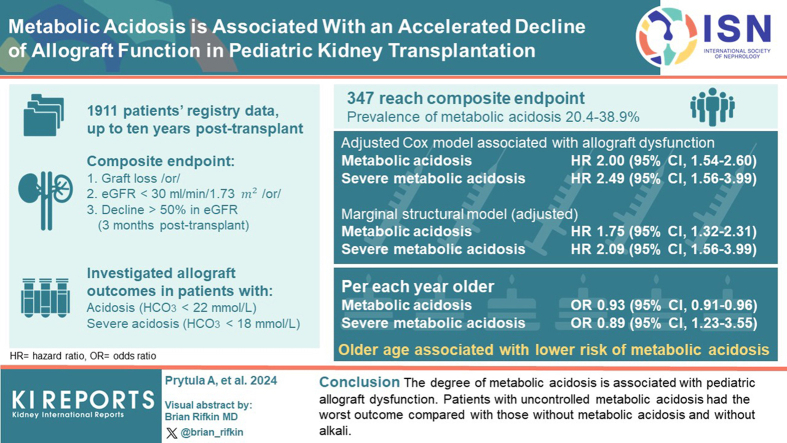

Metabolic acidosis defined as a HCO3^−^ concentration < 22 mmol/l is common in children with pretransplant chronic kidney disease (CKD). In the Cardiovascular Comorbidity in Children with CKD Study of 704 participants, 43%, 61%, and 45% of children with CKD stages 3, 4, and 5, respectively, had metabolic acidosis.^1^ In addition, patients with HCO_3_^−^ < 18 mmol/l had worse 5-year kidney survival, defined as either the need for kidney replacement therapy or a 50% decline in the eGFR at enrollment; 53% as compared with 78% in those without metabolic acidosis and 75% in those with HCO_3_^−^ between 18 and 22 mmol/l. Higher CKD stage and congenital anomalies of the kidney and urinary tract were associated with a higher prevalence of metabolic acidosis, whereas the use of loop diuretics and peritoneal dialysis as compared to hemodialysis were associated with a lower likelihood of metabolic acidosis.^1^ Although successful KTx largely corrects some risk factors underlying metabolic acidosis such as low eGFR, it may also introduce new ones, such as calcineurin inhibitor-based immunosuppression.^2^

In adult KTx recipients, metabolic acidosis has a higher prevalence and severity than pretransplant cohorts with matched CKD stages.^3^ Complications of metabolic acidosis include anemia, bone disease, protein catabolism, and growth failure, and are associated with inferior patient-outcomes and graft outcomes.^4^ In a retrospective multicenter study of 2318 adult KTx recipients, metabolic acidosis was associated with increased mortality, graft failure, and death-censored graft failure, even after adjustment for confounding eGFR.^5^ In another observational study, patients with HCO_3_^−^ < 24 mmol/l at 1 year posttransplant had an increased risk of cardiovascular events and all-cause mortality.^6^ Pediatric data on the risk factors and association of metabolic acidosis with allograft outcome are limited. Metabolic acidosis was present in approximately 30% of pediatric transplant recipients and showed an inverse association with body height, leg length, and sitting height.^7^^,^^8^

Another question is whether correction of metabolic acidosis with alkali supplementation improves graft outcome. In a recent randomized prospective study of adult KTx recipients with mild metabolic acidosis, correction of metabolic acidosis did not result in a slower decline in eGFR over 2 years.^9^ Given the differences in age, comorbidities, comedication, and diet between pediatric and adult KTx recipients, these findings should not be directly extrapolated to a pediatric population. In addition, the determinants and clinical consequences of metabolic acidosis have not yet been investigated in a large cohort of pediatric KTx recipients and may differ from those in adults. Because hard end points such as mortality or graft failure are rare in children, long-term data on the association between metabolic acidosis and graft outcome in pediatric KTx recipients are lacking. Furthermore, it is difficult to determine whether this association is merely a consequence of the declining kidney function or whether metabolic acidosis independently contributes to a more rapid decline in allograft function.

Therefore, the primary objective of our study was to analyze the relationship between time-varying metabolic acidosis and allograft outcome in a large European cohort of pediatric kidney transplant recipients. Using marginal structural models, we aimed to estimate the effect of metabolic acidosis on allograft dysfunction.^10^ Furthermore, we aimed to analyze the evolution of metabolic acidosis after KTx and to identify its clinical and biochemical determinants at 3 months posttransplant. Finally, we investigated whether alkali supplementation was associated with amelioration of metabolic acidosis and improved allograft outcome.

## Methods

### Patients and Follow-up

This retrospective, multicenter, longitudinal cohort study included pediatric KTx recipients younger than 19 years at the time of KTx enrolled in the Cooperative European Paediatric Renal Transplant Initiative Registry (CERTAIN). Patients who experienced graft failure or died within 3 months posttransplant were excluded.

The CERTAIN registry collects detailed longitudinal clinical and laboratory data and applies rigorous validity checking procedures (http://www.certain-registry.eu/). Participation in the CERTAIN registry is approved by the ethics committee at each center. Informed consent was obtained from the parents or legal guardians before enrollment, with assent from patients when appropriate for their age. The time points of data collection and the corresponding time intervals were as follows: baseline (pretransplant), at months 1, 3, 6, 9, 12, and every 6 months thereafter up to 10 years posttransplant; Details are in the Description of the CERTAIN registry: completeness and quality of data in the Supplementary Material. All procedures and immunosuppressive regimens were performed according to local institutional protocols. Anthropometric, clinical, and biochemical data were collected as part of a routine follow-up at each center. Z-scores for body mass index, weight, height, and blood pressure (BP) were calculated according to normative charts.^11^ Sodium bicarbonate was used as the only alkali therapy.

The study was performed in accordance with the Declaration of Helsinki and the Declaration of Istanbul on Organ Trafficking and Transplant Tourism. The study was designed, analyzed, and reported according to the STROBE guidelines (https://www.strobe-statement.org).

### Laboratory Measurements and Definitions

Serum creatinine and HCO_3_^−^ were measured locally, and data were reported to the CERTAIN registry. In accordance with previous reports, metabolic acidosis was defined as severe HCO_3_^−^ ≤ 18 mmol/l), mild to moderate (HCO_3_^−^: 18.1–21.9 mmol/l), or no acidosis (HCO_3_^−^ ≥ 22 mmol/l).^1^ eGFR was calculated using the 2009 revised bedside Schwartz formula.^12^

### Statistical Analysis

Statistical analysis was performed with R Statistical Software version 4.2.2 (Vienna, Austria). Continuous variables with normal distribution were reported as mean ± SD, whereas median with interquartile range was reported for skewed variables. Normality was assessed using normality tests and QQ plots. Categorical variables were reported as numbers (*n*) and percentages (%). Because graft loss is a rare event in pediatric KTx recipients, we used a composite end point called allograft dysfunction as the primary outcome measure, defined as either graft loss, or eGFR ≤30 ml/min per 1.73 m^2^ or a ≥50% decline from baseline eGFR at 3 months posttransplant, whichever occurred first. In 119 patients, eGFR at 3 months posttransplant was set at 120 ml/min per 1.73 m^2^.

We performed a survival analysis examining the associations between time-varying metabolic acidosis and time to composite end point. Because data were available in follow-up time intervals, it was assumed that time-dependent covariates change at the beginning of an interval whereas the event occurs at the end of an interval. First, survival probabilities for allograft dysfunction with HCO_3_^−^ categories as a time-varying exposure were visualized using Kaplan-Meier extended methods, including the HRs with 95% CIs.

Next, unadjusted Cox regression analyses stratified by center were performed to assess the association between metabolic acidosis (HCO_3_^−^ < 22 mmol/l), severe metabolic acidosis (HCO_3_^−^ ≤ 18 mmol/l), and time to composite end point. Conventional center-stratified–extended Cox models were then fitted to estimate the independent association between time-varying metabolic acidosis and allograft dysfunction, after adjustment for potential confounders, including the time-varying covariates, allograft rejection and systolic BP Z-score quartiles. Proportional hazards assumption and linearity were assessed for each covariate before inclusion in the model. In the case of nonlinearity, a spline was fitted and stratified analyses were performed. The association between time-varying metabolic acidosis and allograft dysfunction was further tested using marginal structural models. Therefore, we applied the inverse probability of weights to construct a pseudo-population in which the exposure variable was not confounded by the time-varying confounder eGFR and other outcome-related covariates, thereby providing a more accurate estimate of the independent association between metabolic acidosis and outcome;^13^, ^14^, ^15^, ^16^ details are described in Statistical methods- structural marginal models” in the Supplementary Material.

Secondary analyses included identification of risk factors associated with metabolic acidosis at 3 months posttransplant. Risk factors for metabolic acidosis at 3 months after transplantation were examined using logistic regression with HCO_3_^−^ < 22 mmol/l and HCO_3_^−^ ≤ 18 mmol/l as outcome variables. Finally, the association between alkali supplementation at the preceding time point, HCO_3_^−^ at 1 year posttransplant and time to composite end point was visualized using Kaplan-Meier curve. *Post hoc* Tukey tests were used to calculate HRs with 95% CIs to compare the groups. Differences between patients in whom alkali improved metabolic acidosis and those in whom it did not were analyzed by logistic regression.

## Results

### Cohort Characteristics and Incidence of Death, Graft Loss, and Allograft Dysfunction

Data on 2342 grafts in patients younger than 19 years were reported to the CERTAIN registry. Six children died and 31 experienced graft loss within 3 months posttransplant and were excluded from the analyses. We included 1911 patients from 49 centers in 17 countries who underwent KTx between September 1993 and April 2021 and had at least 1 documented HCO_3_^−^ (see also “Patient inclusion per country” and “10 centers with the highest number of reported patients” in the Supplementary Material). One graft per patient was analyzed. Patient characteristics are shown in Table 1.

**Table 1:** Patient and transplant characteristics

Baseline characteristics	Patient cohort (*N* = 1911)
Recipient age, yr	9.92 (Q1: 4.92; Q3: 14.08)
Male sex, *n* (%)	1162 (60.8)
Weight, kg	26.00 (Q1: 15.70; Q3: 41.80)
Weight Z-score	−1.52 (Q1: −2.55; Q3: −0.61)
Height Z-score	−1.93 (Q1: −3.10; Q3: −1.03)
BMI Z-score	−0.4 (Q1: −1.23; Q3: 0.46)
Primary kidney disease, *n* (%)	
CAKUT	855 (44.7)
Glomerulopathy	430 (22.5)
Tubulointerstitial nephritis and cystic kidney disease	385 (20.1)
HUS	85 (4.4)
Vascular complications	46 (2.4)
Other/unknown	110 (5.8)
Dialysis mode, *n* (%)	
None (preemptive KTx)	421 (22)
Peritoneal dialysis	798 (41.8)
Hemodialysis	689 (36.1)
Unknown	3 (0.2)
Dialysis vintage, mo	18 (Q1: 8; Q3: 31)
Disease vintage, mo	63 (Q1: 31; Q3: 125)
Decade of KTx, *n* (%)	
Before the year 2000	21 (1.1)
Between 2001 and 2010	367 (19.2)
After the year 2010	1523 (79.7)
KTx, *n* (%)	
First	1704 (89.2)
Second	205 (10.7)
Third	2 (0.1)
Donor source, *n* (%)	
Deceased	1293 (67.7)
Living-related	612 (32.0)
Living-unrelated	6 (0.3)
Delayed graft function, *n* (%)	140 (7.3)
Initial immunosuppressive therapy, *n* (%)	
Glucocorticoids	1802 (94.3)
Calcineurin inhibitors	1471 (76.9)
Tacrolimus	1434 (75.0)
Mycophenolate mofetil	1528 (80.0)

BMI, body mass index; CAKUT, congenital anomalies of the kidney and urinary tract; HUS, hemolytic uremic syndrome; KTx, kidney transplantation; Q, quartile.

Data are presented as number *n* (%) or mean (SD) for normally distributed variables or as median and first (Q1) and third (Q3) quartile for skewed variables.

Eighteen (0.9%) patients died between 3 months and 10 years of follow-up. After 1, 3, 5, and 10 years of follow-up, data were available for 1787 (93.5%), 1183 (61.9%), 698 (36.5%), and 158 (8.2%) of the patients, respectively. The median follow-up period was 2 years. The total number of documented measurements was 19,658, and HCO_3_^−^ was not reported in 5156 (26.2%) intervals. Three hundred forty-seven patients (18.1%) reached the composite end point: *n* = 16 graft failure, *n* = 178 eGFR < 30 ml/min per 1.73 m^2^, *n* = 153 >50% decline from baseline eGFR at month 3 posttransplant. Forty-nine patients experienced graft failure after the composite end point had been reached.

### Evolution and Determinants of Metabolic Acidosis

**Figure 1:**
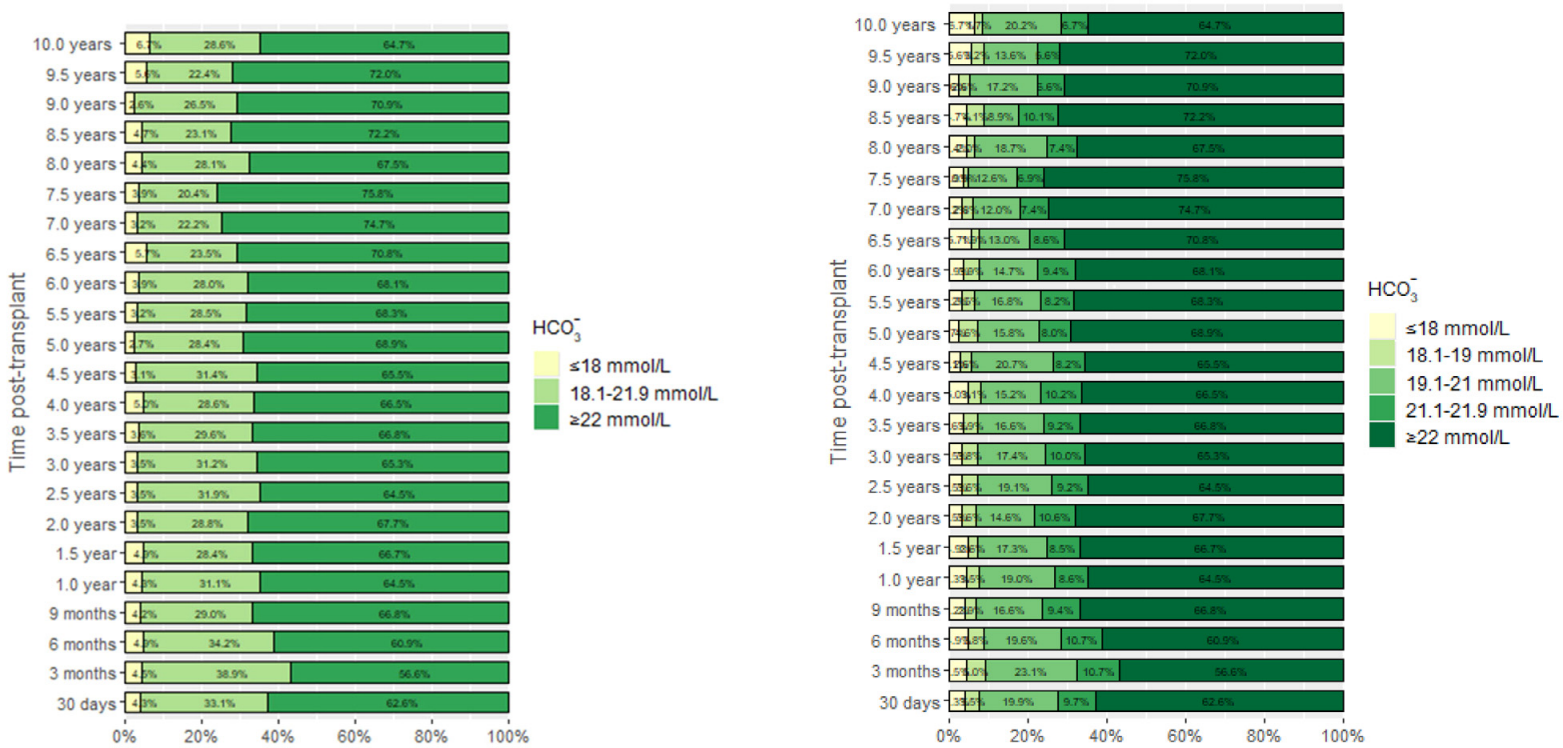
(a) Proportion of patients with severe metabolic acidosis (HCO3⁻ ≤ 18 mmol/l, yellow bars), mild-to-moderate metabolic acidosis (HCO3⁻, 18.1–21.9 mmol/l, light green bars) and no metabolic acidosis (HCO3⁻ ≥22 mmol/l, dark green bars). (b) As above, with additional breakdown of mild-to-moderate metabolic acidosis into subgroups (HCO3⁻, 18.1–19 mmol/l; HCO3⁻, 19.1–21 mmol/l; and HCO3⁻, 21.1–21.9 mmol/l). HCO3⁻, serum bicarbonate concentration.

As shown in Figure 1, the proportion of patients with mild-to-moderate metabolic acidosis and severe metabolic acidosis ranged from 20.4% to 38.9% and from 2.7% to 6.7%, respectively, over time. The median plasma bicarbonate levels in stable kidney transplant recipients beyond 3 months from transplantation before the year 2001, between 2001 and 2010, and after 2010 were 23 mmol/l, 22.9 mmol/l, and 22.9 mmol/l. The respective percentage of time points with alkali supplementation were 28, 26.5, and 32.7. As shown in Table 2, older patient age was associated with a lower risk of both metabolic acidosis (OR, 0.93/yr older; 95% CI, 0.91–0.96; *P* < 0.001) and severe metabolic acidosis (OR, 0.89; 95% CI, 0.84–0.95; *P* = 0.001) at 3 months posttransplant. A higher proportion of patients under alkaline therapy had HCO_3_^−^ < 22 mmol/l (OR, 1.40; 95% CI, 1.06–1.86; *P* = 0.020) and HCO_3_^−^ ≤ 18 mmol/l (OR, 2.34; 95% CI, 1.31–4.16; *P* = 0.004) compared to the cohort that did not require alkaline therapy. Live donor KTx was associated with a lower risk of metabolic acidosis (OR, 0.69; 95% CI, 0.52–0.91; *P* = 0.009). The lowest BP Z-score quartile (OR, 3.99; 95% CI, 1.64–10.08; *P* = 0.003), eGFR (OR, 0.99; 95% CI, 0.98–1.00; *P* = 0.030) and tacrolimus predose concentration (OR, 1.16; 95% CI, 1.06–1.27; *P* = 0.001) were determinants of severe metabolic acidosis at month 3 posttransplant.

**Table 2:** Determinants of metabolic acidosis and severe metabolic acidosis at month 3 posttransplant

	HCO3⁻ < 22 mmol/l		HCO3⁻ ≤ 18 mmol/l	
Variable	Univariable: OR (95% CI); *P* value	Multivariable: OR (95% CI); *P* value	Univariable: OR (95% CI); *P* value	Multivariable: OR (95% CI); *P* value
Recipient age (yr)	0.96 (0.94–0.98); *P* < 0.001	0.93 (0.91–0.96); *P* < 0.001	0.93 (0.89–0.98); *P* = 0.006	0.89 (0.84–0.95); *P* = 0.001
Female sex	1.14 (0.92–1.40); *P* = 0.223	1.31 (1.0–1.72); *P* = 0.048	1.65 (1.02–2.69); *P* = 0.041	2.15 (1.21–3.86); *P* = 0.010
eGFR (ml/min per 1.73 m^2^)	1.00 (0.99–1.00); *P* = 0.207	1.00 (0.99–1.00); *P* = 0.069	0.99 (0.98–1.00); *P* = 0.067	0.99 (0.98–1.00); *P* = 0.030
Alkali supplementation (yes)	2.02 (1.60–2.55); *P* < 0.001	1.40 (1.06–1.86); *P* = 0.020	2.56 (1.57–4.17); *P* < 0.001	2.34 (1.31–4.16); *P* = 0.004
Allograft rejection (yes)	1.26 (0.82–1.95); *P* = 0.287	1.16 (0.68–1.97); *P* = 0.581	0.99 (0.30–2.47); *P* = 0.988	0.56 (0.13–1.72); *P* = 0.310
BMI Z-score	0.98 (0.90–1.07); *P* = 0.673	0.97 (0.87–1.09); *P* = 0.651	0.92 (0.75–1.13); *P* = 0.398	0.88 (0.69–1.13); *P* = 0.567
Decade of KTx (after 2010)	1.37 (1.07–1.77); *P* = 0.014	1.06 (0.72–1.55); *P* = 0.779	1.19 (0.66–2.29); *P* = 0.584	1.47 (0.63–4.06); *P* = 0.410
Tacrolimus predose concentration	1.03 (0.98–1.08); *P* = 0.212	1.02 (0.97–1.08); *P* = 0.348	1.14 (1.05–1.24); *P* = 0.002	1.16 (1.06–1.27); *P* = 0.001
Systolic BP Z-score				
Q2–Q3	Reference	Reference	Reference	Reference
Q1	1.10 (0.76–1.58); *P* = 0.613	0.88 (0.57–1.34); *P* = 0.544	3.04 (1.36–7.01); *P* = 0.007	3.99 (1.64–10.08); *P* = 0.003
Q4	0.82 (0.65–1.05); *P* = 0.113	0.81 (0.60–1.08); *P* = 0.150	1.59 (0.84–3.27); *P* = 0.175	1.93 (0.94–4.30); *P* = 0.087
Time on dialysis	1.01 (1.00–1.01); *P* = 0.002	1.00 (1.00–1.01); *P* = 0.245	1.01 (1.00–1.02); *P* = 0.044	1.01 (0.99–1.02); *P* = 0.274
Primary diagnosis				
CAKUT	Reference	Reference	Reference	Reference
Glomerulopathy	0.97 (0.75–1.25); *P* = 0.803	0.83 (0.60–1.14); *P* = 0.252	0.87 (0.45–1.59); *P* = 0.655	0.87 (0.42–1.75); *P* = 0.708
Tubulointerstitial disease	0.73 (0.55–0.97); *P* = 0.028	0.62 (0.44–0.87); *P* = 0.007	1.10 (0.58–2.00); *P* = 0.758	1.13 (0.53–2.32); *P* = 0.738
HUS	0.94 (0.56–1.55); *P* = 0.799	1.03 (0.54–1.95); *P* = 0.935	0.61 (0.10–2.07); *P* = 0.505	0.84 (0.13–3.14); *P* = 0.819
Other or unknown	0.62 (0.38–1.00); *P* = 0.055	0.52 (0.27–0.94); *P* = 0.035	0.77 (0.18–2.22); *P* = 0.673	1.27 (0.28–4.07); *P* = 0.714
Donor source (living related)	0.64 (0.51–0.80); *P* <0.001	0.69 (0.52–0.91); *P* = 0.009	0.55 (0.29–0.96); *P* = 0.047	0.85 (0.42–1.64); *P* = 0.638

BMI, body mass index; BP, blood pressure; CAKUT, congenital anomalies of the kidney and urinary tract; CI, confidence interval; eGFR, estimated glomerular filtration rate; HUS, hemolytic uremic syndrome; KTx, kidney transplantation; OR, odds ratio; Q quartile (Q1 denotes the lowest BP Z-score).

### Association Between Metabolic Acidosis and Allograft Dysfunction

**Figure 2:**
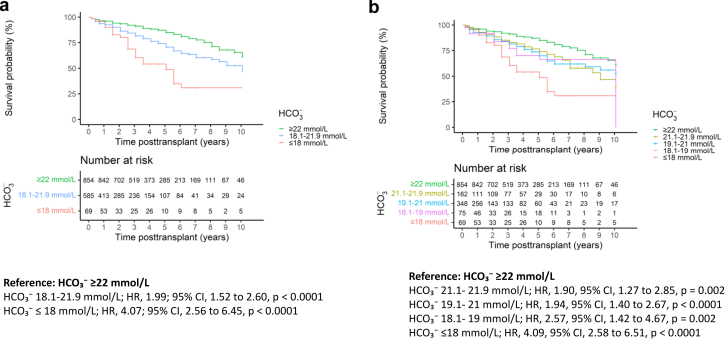
(a) Association between the cumulative incidence of time to composite end point and time-varying severe metabolic acidosis (red line), mild-to-moderate metabolic acidosis (blue line), and no acidosis (green line). (b) Association between the degree of time-varying metabolic acidosis and time to composite end point. Number at risk in Figure 2a and b corresponds to the number of patients with available HCO₃⁻ at a given time point. CI, confidence interval; HCO3⁻, serum bicarbonate concentration; HR, hazard ratio.

The unadjusted survival probabilities for allograft dysfunction with the time-varying covariates HCO_3_^−^ < 22 mmol/l (HR, 1.99; 95% CI, 1.52–2.60; *P* < 0.001) and HCO_3_^−^ ≤ 18 mmol/l (HR, 4.07; 95% CI, 2.56–6.45; *P* < 0.0001) were associated with time to composite end point (Figure 2a). The association between the degree of time-varying metabolic acidosis and time to composite end point is shown in Figure 2b. Mild metabolic acidosis with HCO_3_^−^ levels of 21.1 to 21.9 mmol/l was associated with time to composite end point (HR, 1.90, 95% CI, 1.27–2.85; *P* = 0.002), and there was a stepwise decrease in the probability of having no graft dysfunction (free from the composite end point) with a higher degree of metabolic acidosis. To verify the association between time-varying metabolic acidosis and allograft dysfunction, extended Cox models were applied and compared with marginal structural models. This analysis included 1872 patients with 10 years of follow-up, of whom 262 patients reached the composite end point. As shown in Table 3, both time-varying metabolic acidosis and severe metabolic acidosis were independently associated with time to composite end point. Extended Cox models adjusted for known risk factors for allograft dysfunction showed HR, 2.00 (95% CI, 1.54–2.60; *P* < 0.001) for metabolic acidosis and HR, 2.49 (95% CI, 1.56–3.99; *P* < 0.001) for severe metabolic acidosis. The association of metabolic acidosis (HR, 1.75; 95% CI, 1.32–2.31; *P* < 0.001) and severe metabolic acidosis (HR, 2.09; 95% CI, 1.23 to 3.55; *P* = 0.006) with graft dysfunction was confirmed in the marginal structural model, suggesting that their association with allograft dysfunction is independent of time-varying eGFR and other confounders included in the inverse probability of weights model. The fully adjusted marginal structural models for assessing the association of metabolic acidosis and severe metabolic acidosis with allograft dysfunction are shown in Table 4.

**Table 3:** Comparison between the conventional Cox proportional hazards models and the marginal structural models for assessing the association of time-varying metabolic acidosis and severe metabolic acidosis with allograft dysfunction

Cox model (unadjusted)	HR	95% CI	*P* value
Metabolic acidosis (HCO_3_^−^ < 22 mmol/l)	2.19	1.69–2.84	<0.001
Severe metabolic acidosis (HCO_3_^−^ ≤ 18 mmol/l)	3.11	2.01–4.81	<0.001
Cox model (adjusted)			
Metabolic acidosis (HCO_3_^−^ < 22 mmol/l)	2.00	1.54–2.60	<0.001
Severe metabolic acidosis (HCO_3_^−^ ≤ 18 mmol/l)	2.49	1.56–3.99	<0.001
Marginal structural model (unadjusted)			
Metabolic acidosis (HCO_3_^−^ <22 mmol/l)	1.83	1.39–2.40	<0.001
Severe metabolic acidosis (HCO_3_^−^ ≤ 18 mmol/l)	2.25	1.32–3.84	<0.001
Marginal structural model (adjusted)			
Metabolic acidosis (HCO_3_^−^ < 22 mmol/l)	1.75	1.32–2.31	<0.001
Severe metabolic acidosis (HCO_3_^−^ ≤ 18 mmol/l)	2.09	1.23–3.55	0.006

CI, confidence interval; eGFR, estimated glomerular filtration rate; HR, hazard ratio; KTx, kidney transplantation.

The Cox proportional hazards models were adjusted for baseline and time-varying risk factors for allograft dysfunction (baseline confounders: primary kidney disease, sex, recipient age at KTx, donor source, decade of transplantation, dialysis vintage, graft sequence, body mass index Z-score at KTx, total number of human leukocyte antigen mismatches and delayed graft function stratified within and beyond the first year posttransplant; time-varying confounders: allograft rejection and systolic blood pressure Z-score categorized by quartiles).

For marginal structural models the inverse probability of weights model was constructed, including the time-varying confounder eGFR. Models were then adjusted for other potential risk factors for allograft dysfunction.

**Table 4:** Full marginal structural models to assess of the association of metabolic acidosis and severe metabolic acidosis with allograft dysfunction, adjusted for baseline and time-varying risk factors for allograft dysfunction

Marginal structural model (adjusted)	HCO_3_^−^ < 22 mmol/l			HCO_3_^−^ ≤ 18 mmol/l		
	HR	95% CI	*P* value	HR	95% CI	*P* value
Metabolic acidosis (exposure)	1.75	1.32–2.31	<0.001	2.09	1.23–3.55	0.006
Acute rejection (time-varying)	8.71	6.17–12.31	<0.001	9.99	7.15–13.98	<0.001
Systolic BP Z-score Q1 (time-varying)	0.98	0.63–1.52	0.918	1.16	0.77–1.77	0.478
Systolic BP Z-score Q4 (time-varying)	1.50	1.11–2.04	0.008	1.50	1.10–2.05	0.01
Sex (female)	0.83	0.62–1.11	0.210	0.87	0.66–1.16	0.346
Age at KTx	0.98	0.95–1.01	0.139	0.98	0.95–1.01	0.111
Body mass index Z-score at KTx	0.85	0.76–0.95	0.005	0.87	0.78–0.98	0.017
Donor source (living)	1.09	0.82–1.46	0.550	1.12	0.84–1.50	0.433
Transplant sequence	1.06	0.66–1.71	0.811	1.00	0.61–1.64	0.998
Year of KTx (beyond 2010)	0.90	0.65–1.25	0.523	0.83	0.59–1.16	0.277
Primary kidney disease: glomerular	0.82	0.58–1.16	0.264	0.91	0.65–1.29	0.606
Primary kidney disease: tubulointerstitial	1.02	0.71–1.46	0.911	1.05	0.74–1.50	0.769
Primary kidney disease: HUS	0.85	0.45–1.61	0.618	0.87	0.45–1.67	0.671
Primary kidney disease: vascular complications	1.48	0.70–3.11	0.303	1.57	0.81–3.07	0.184
Primary kidney disease: other/unknown	0.66	0.37–1.21	0.181	0.67	0.35–1.26	0.208
Number of HLA-MM	0.99	0.90–1.11	0.931	0.98	0.89–1.10	0.809
Dialysis vintage (mo)	1.00	0.99–1.01	0.877	1.00	0.99–1.01	0.737
Delayed graft function (association with allograft dysfunction within the first year posttransplant)	1.12	0.48–2.62	0.794	1.1	0.49–2.49	0.819
Delayed graft function (association with allograft dysfunction beyond 1 year posttransplant)	1.29	0.81–2.06	0.298	1.34	0.82–2.19	0.240

BP, blood pressure; CI, confidence interval; HLA-MM, human leukocyte antigen mismatch; HR, hazard ratio; HUS, hemolytic uremic syndrome; KTx, kidney transplantation; Q1, first quartile (lowest BP); Q4, fourth quartile (highest BP).

We also performed a sensitivity analysis of the association between time-varying metabolic acidosis and time to composite end point using only bicarbonate levels with a corresponding normal anion gap. The results were in line with those shown above (see “Sensitivity analysis- association between time-varying metabolic acidosis and time to composite end point using only bicarbonate levels with a corresponding normal anion gap,” Supplementary Figures S1 and S2 in the Supplementary Material).

### Improvement in Metabolic Acidosis With Alkali Supplementation and Allograft Dysfunction

**Figure 3:**
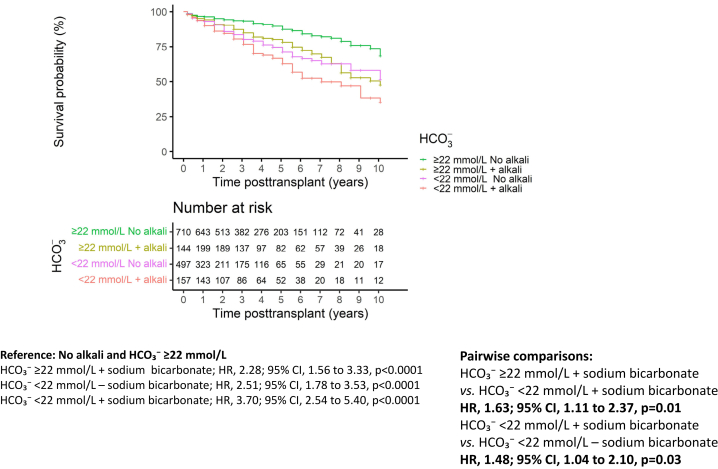
Association between the cumulative incidence of time to composite end point and the combination of time-varying HCO₃⁻ and alkali supplementation at the previous time point. Number at risk corresponds to the number of patients with available HCO₃⁻ at a given time point and information on alkali supplementation at the previous time point. CI, confidence interval; HCO3⁻, serum bicarbonate concentration; HR, hazard ratio.

To analyze the association of alkali supplementation with allograft dysfunction we performed a survival analysis with time to composite end point in patients with time-varying HCO_3_^−^ ≥ or <22 mmol/l and with or without alkali supplementation at the previous time interval. As shown in Figure 3, patients with HCO_3_^−^ < 22 mmol/l or those with well-controlled metabolic acidosis under alkali supplementation had a higher risk of allograft dysfunction than patients without metabolic acidosis and without alkali supplementation (reference). Patients with uncontrolled metabolic acidosis on alkali supplementation had the worst outcome compared with the reference group (HR, 3.70; 95% CI, 2.54–5.40). Patients in whom alkali supplementation improved metabolic acidosis had better outcome than those with uncontrolled metabolic acidosis (HR, 1.63; 95% CI, 1.11–2.37). Finally, patients with uncontrolled metabolic acidosis on alkali therapy had a worse outcome than those with HCO_3_^−^ <22 mmol/l and no alkali therapy (HR, 1.48; 95% CI, 1.04–2.10).

We then examined whether there were differences in age, sex, dose of sodium bicarbonate in mg/kg body weight, and eGFR between patients who achieved adequate control of metabolic acidosis on alkali supplementation and those who did not. We found that among children who were taking alkali at month 9 posttransplant, those with uncontrolled metabolic acidosis at year 1 (*n* = 152, 42%) were significantly younger (OR, 1.07/yr younger; 95% CI, 1.02–1.1; *P* = 0.01). The analysis of factors associated with improvement of metabolic acidosis with alkali supplementation is shown in Supplementary Table S1.

## Discussion

To our knowledge, this is the first study to identify time-varying metabolic acidosis as an independent risk factor for allograft dysfunction in a large cohort of pediatric KTx recipients. The association between metabolic acidosis and allograft dysfunction was estimated by using both the conventional Cox models and the marginal structural models. In the latter, covariates affecting both the outcome and time-varying exposure, such as eGFR, were included in the inverse probability of weights model. In the case of a reciprocal relationship between a time-varying exposure (e.g., metabolic acidosis) and a time-varying confounder (e.g., eGFR), conventional Cox proportional hazards models may yield biased estimates of the exposure-outcome association. In this study, both the marginal structural models and the conventional Cox proportional hazards models showed an association between metabolic acidosis and allograft outcome, suggesting that it is independent of eGFR.

Metabolic acidosis impairs kidney function by increasing levels of angiotensin II, aldosterone, and endothelin-1, leading to inflammation and fibrosis.^17^^,^^18^ Ongoing ammoniagenesis exacerbates the damage through complement activation and deposition.^19^ In patients with CKD, metabolic acidosis is associated with accelerated loss of kidney function.^20^^,^^21^ In addition, a large observational study of adult KTx recipients reported an association between metabolic acidosis, graft failure, and mortality. There is also evidence suggesting that treatment of chronic metabolic acidosis in patients with CKD reduces inflammation and fibrosis.^18^^,^^21^ Therefore, a randomized trial was conducted to analyze the effect of sodium bicarbonate supplementation on the rate of eGFR decline in 240 adult KTx recipients with a mean HCO_3_^−^ level of 21 mmol/l (placebo group) to 21.3 mmol/l (treatment group), which is mild metabolic acidosis.^9^ There was no difference in eGFR decline after 2 years of follow-up, and the authors concluded that treatment with sodium bicarbonate should not be recommended in adult KTx recipients with metabolic acidosis to preserve allograft function.^9^ These findings may not be applicable to pediatric KTx recipients. We report a stepwise association of the degree of metabolic acidosis with allograft dysfunction. Only a small proportion of children with mild metabolic acidosis (e.g., HCO_3_^−^, 21 mmol/L) experienced allograft dysfunction. In view of our findings, the results of the prospective study in adult kidney transplant recipients cannot be extrapolated to pediatric patients. Furthermore, we report that as many as 42% of pediatric patients in a real-world setting did not achieve sufficient control of metabolic acidosis under alkali treatment. To the best of our knowledge, this has not been reported in adults and may be associated with young patient age. We demonstrated distinct risk factors for metabolic acidosis in pediatric KTx recipients such as younger patient age, which is consistent with a recent report in a cohort of 63 pediatric KTx recipients.^22^ Several studies have shown that young children require higher doses of tacrolimus to achieve target exposure.^23^^,^^24^ Tacrolimus affects key transport proteins that are involved in acid-base homeostasis in the proximal and distal tubules, including endothelin-1 and H(+)-ATPase transport protein.^25^ This dose-dependent tubular toxicity may be reversed by reducing the dose of tacrolimus.^25^ These considerations are supported by the fact that in our study, tacrolimus predose concentration was associated with severe metabolic acidosis, along with a low systolic BP Z-score. Salt and bicarbonate wasting associated with low systolic BP are common in children with congenital anomalies of the kidney and urinary tract and may still be clinically relevant at 3 months posttransplant.

We show that 42% of children did not respond to the alkaline therapy. Although treatment failure may be due in part to poor therapy adherence, other factors may also play a role. Experimental data suggest that calcineurin inhibitors impair mineralocorticoid transcriptional activity in the distal tubular cells and may cause aldosterone resistance, hyperkalemia, and metabolic acidosis, also known as type IV metabolic acidosis.^26^ In a cohort of 576 adult KTx recipients with stable allograft function, 28% developed type IV metabolic acidosis, which may respond to treatment with fludrocortisone rather than alkali supplementation.^27^ Differentiating between different types of metabolic acidosis was beyond the scope of our study, but we did show that young age, and therefore higher tacrolimus dose requirements, were associated with inadequate control of metabolic acidosis with alkali supplementation.

The strengths of our study include the multicenter design, which allowed for reliable statistical analysis in the largest cohort of pediatric KTx recipients to date with 10-year follow-up. Data from the academic CERTAIN registry closely reflect real-world clinical practice.^28^ Our study also has several limitations. Because this is a retrospective registry analysis, we cannot establish causality, only association. We cannot exclude residual confounding by factors not reported in the registry and therefore not included in the statistical analysis, such as donor profile, proteinuria, the presence of donor-specific antibodies, income, or educational level which may impact on care and diet.^29^ The observation that patients treated with alkali therapy who normalized their bicarbonate levels remain at higher risk of allograft dysfunction suggests that acidosis or its treatment are associated with another risk factor of allograft dysfunction that may not be accounted for.

In conclusion, in this observational cohort study of pediatric KTx recipients, we identified a stepwise association of metabolic acidosis and allograft dysfunction. Young age was associated with metabolic acidosis and failure of alkaline therapy. Prospective studies are needed to analyze the potential causal relationship between different degrees of metabolic acidosis and allograft dysfunction, to define target HCO_3_^−^ levels, and to establish personalized management strategies.

## Appendix

Transplantation Working Group Member List: Gema Ariceta, Atif Awan, Sevcan Bakkaloğlu, Marjolein Bonthuis, Charlotte Bootsma Robroeks, Antonia Bouts, Martin Christian, Marlies Cornelissen, Ali Duzova, Nasrin Esfandiar, Luciana Ghio, Ryszard Grenda, Isabella Guzzo, Maria Herrero Goni, Julien Hogan, Nattaphorn Hongsawong, Nele Kanzelmeyer, Aysun Karabay Bayazit, Gülşah Kaya Aksoy, Noel Knops, Linda Koster Kamphuis, Daniella Levy Erez, Victor Lopez-Baez, Alvaro Madrid, Stephen Marks, Anette Melk, Luisa Murer, Lars Pape, Licia Peruzzi, Edita Petrosyan, Evgenia Preka, Nikoleta Printza, Andreea Liana Rachisan, Ann Raes, Mohan Shenoy, Oguz Soylemezoglu, Luca Dello Strologo, Ana Teixeira, Rezan Topaloglu, Markus Weitz, Jakub Zieg, Galia Zlatanova, Christian Patry, Jerome Harambat.

CKD Mineral and Bone Disorder (CKD-MBD) Working Group Member List: Ayşe Ağbaş, Varvara Askiti, Marina Avramescu, Justine Bacchetta, Sevcan Bakkaloglu, Marjolein Bontuis, Caroline Booth, Laurene Dehoux, Giacomo Dizazzo, Dorota Drozdz, Ismail Dursun, Michaela Gessner, Jaap Groothoff, Giuliana Guido, Isabella Guzzo, Aysun Karabay Bayazit, Guenter Klaus, Linda Koster-Kamphuis, Alexander Lalayiannis, Maren Leifheit-Nestler, Sinha Manish, Chiara Matteucci, Jun Oh, Ozan Ozkaya, Edita Petrosyan, Christine Pietrement, Agnieszka Prytula, George Reusz, Franz Schaefer, Claus Peter Schmitt, Anne Schön, Fatma Lale Sever, Stella Stabouli, Serra Sürmeli Döven, Camilla Tondel, Enrico Verrina, Enrico Vidal, Dean Wallace, Zainab Arslan.

CERTAIN Research Network: M. Bald (Stuttgart), H. Fehrenbach (Memmingen), D. Haffner (Hannover), M. Hansen (Frankfurt), C. Hempel (Leipzig), U. John (Jena), G. Klaus (Marburg), J. König (Münster), B. Lange-Sperandio (Munich), D. Müller (Berlin), J. Oh (Hamburg), L. Pape (Essen), M. Pohl (Freiburg), K. Sauerstein (Erlangen), G. Schalk (Bonn), H. Staude (Rostock), P. Strotmann (Munich TUM), LT Weber (Cologne), M. Weitz (Tübingen), L. Berta (Budapest, Hungary), and K. Heindl-Rusai (Vienna, Austria).

## Disclosure

All the authors declared no conflicting interests.

## Supplementary Material

Supplementary File (PDF) **Description of the CERTAIN registry: completeness and quality of data. Statistical methods - structural marginal models. Patient inclusion per country. Ten centers with the highest number of included patients. Sensitivity analysis- association between time-varying metabolic acidosis and time to composite end point using only bicarbonate levels with a corresponding normal anion gap**. **Figure S1.** Association between the cumulative incidence of time to composite end point and time-varying severe metabolic acidosis (red line), mild-to-moderate metabolic acidosis (blue line), and no acidosis (green line) in patients with normal anion gap. **Figure S2.** Association between the degree of time-varying metabolic acidosis and time to composite end point in patients with normal anion gap. **Table S1.** Analysis of factors associated with amelioration of metabolic acidosis under alkali supplementation at 3 months, 1, 3, and 5 years posttransplant.

## Footnotes

Supplementary File (PDF)

**Description of the CERTAIN registry: completeness and quality of data.**

**Statistical methods - structural marginal models.**

**Patient inclusion per country.**

**Ten centers with the highest number of included patients.**

**Sensitivity analysis - association between time-varying metabolic acidosis and time to composite end point using only bicarbonate levels with a corresponding normal anion gap.**
**Figure S1.** Association between the cumulative incidence of time to composite end point and time-varying severe metabolic acidosis (red line), mild-to-moderate metabolic acidosis (blue line), and no acidosis (green line) in patients with normal anion gap. **Figure S2.** Association between the degree of time-varying metabolic acidosis and time to composite end point in patients with normal anion gap. Number at risk in Figures S1 and S2 corresponds to the number of patients with available HCO₃⁻ at a given time point. CI, confidence interval; HR, hazard ratio. **Table S1.** Analysis of factors associated with amelioration of metabolic acidosis under alkali supplementation at 3 months, 1, 3, and 5 years posttransplant.
