# Supplementary material for: Metabolic Acidosis Is Associated With an Accelerated Decline of Allograft Function in Pediatric Kidney Transplantation
Source: Kidney Int Rep. 2024 Apr 8;9(6):1684–93. doi: 10.1016/j.ekir.2024.04.007 (PMC11184248; doi:10.1016/j.ekir.2024.04.007)
Supplement: Supplementary File (PDF) — Description of the CERTAIN registry: completeness and quality of data. Statistical methods - structural marginal models. Patient inclusion per country. Ten centers with the highest number of included patients. Sensitivity analysis- association between time-varying metabolic acidosis and time to composite end point using only bicarbonate levels with a corresponding normal anion gap. Figure S1. Association between the cumulative incidence of time to composite end point and time-varying severe metabolic acidosis (red line), mild-to-moderate metabolic acidosis (blue line), and no acidosis (green line) in patients with normal anion gap. Figure S2. Association between the degree of time-varying metabolic acidosis and time to composite end point in patients with normal anion gap. Table S1. Analysis of factors associated with amelioration of metabolic acidosis under alkali supplementation at 3 months, 1, 3, and 5 years posttransplant. [file mmc1.pdf]

Supplemental material

1. *Description of the CERTAIN registry: completeness and quality of data*

CERTAIN provides a detailed data collection, which allows an in-depth characterization of specific patient cohorts. Data are collected before kidney transplantation, at month 1, 3, 6, 9 and 12 posttransplant, and at 6-month intervals thereafter. In addition, the CERTAIN dataset allows for detailed and flexible documentation of the posttransplant follow-up through continuous entry of any number of relevant data (e.g., laboratory values, drug therapy). Specific case report forms (CRFs) collect detailed and accurate information on relevant data and events of pediatric kidney transplantation in the peri- and posttransplant course. There are two datasets, the minimum required data set and the extended data set. The minimum required dataset is mandatory for all participating centers. The extended data set provides a deeper insight into the clinical course and treatment of patients by documenting additional items, some of which are predefined and some of which can be defined by the participating center. The CERTAIN web application (accessible via http://www.certain-registry.eu/RegApp) has an automatic and manual data validation functionality. During data entry, the data set is automatically validated against predefined plausibility ranges. In addition, the system has an integrated manual quality assurance process. First, documented data must be approved locally at the site; second, a data quality manager at the registry headquarters randomly checks data for plausibility. Only data that pass this quality assurance process are incorporated into the research database. These functions are available anytime, anywhere and require only a standard Web browser and Internet access.

2. *Statistical methods- structural marginal models*

Estimating the effect of an exposure (in our analysis metabolic acidosis) on outcome (composite endpoint of allograft dysfunction) requires adjustment for all risk factors that are also associated with the outcome. When a time-varying confounder is present (eGFR) the estimated effect can be biased and a marginal structural model is preferred instead of regression adjustment (Cole & Hernán 2008, Chesnaye et al. 2022). This approach is ”marginal” (instead of “conditional” in standard adjusted regression) because the patient population is first re-weighted to balance on potential confounders before estimating the effect of interest .

In the present study we applied the inverse probability of weights to construct a pseudo-population in which the exposure variable (metabolic acidosis) was not confounded by time-varying confounder eGFR. Also, we constructed a full weights model including the additional covariates: time-varying rejection, time-varying systolic blood pressure Z-score (spline) , donor source, age at KTx, recipient sex, raft rank, decade of KTx, primary kidney disease, body mass index Z-score, delayed graft function and dialysis vintage. Based on this full weights model we fitted a marginal structural model including only metabolic acidosis given the confounders had been accounted for in the full weights model (Cole & Hernán 2008, Chesnaye et al. 2022).

3. *Patient inclusion per country*

Austria (n=6), Belgium (n=12), Czechia (n=8), France (n=116), Germany (n=857), Greece (n=14), Hungary (n=9), Italy (n=374), Ireland (n=60), Netherlands (n=17), Poland (n=95), Russia (n=18), Slovenia (n=3), Spain (n=34), Switzerland (n=32), Turkey (n=142) and United Kingdom (n=114)

4. 10 centers with the highest number of reported patients

Heidelberg (n=192), Hannover (n=187), Rome (n=172), Padua (n=107), Warsaw (n=95), Manchester (n=93), Essen (n=84), Lyon (n=73) and Hamburg (n=72)

5. Sensitivity analysis- association between time-varying metabolic acidosis and time to composite endpoint using only bicarbonate levels with a corresponding normal anion gap

We had data to calculate anion gap (AG= Na-(Cl+HCO3-)) in 10895 out of 17959 time intervals. Of these, normal anion gap was detected in 8439 intervals. We then performed a sensitivity analysis of the association between time-varying metabolic acidosis and time to composite endpoint using only bicarbonate levels with a corresponding normal anion gap. Survival probabilities for allograft dysfunction with $HCO3^{-}$ categories as time-varying exposure were visualized using the extended Kaplan Meier methods, including the hazard ratio with 95% confidence intervals. The results were in line with those shown in the main manuscript.

6. Figure S1

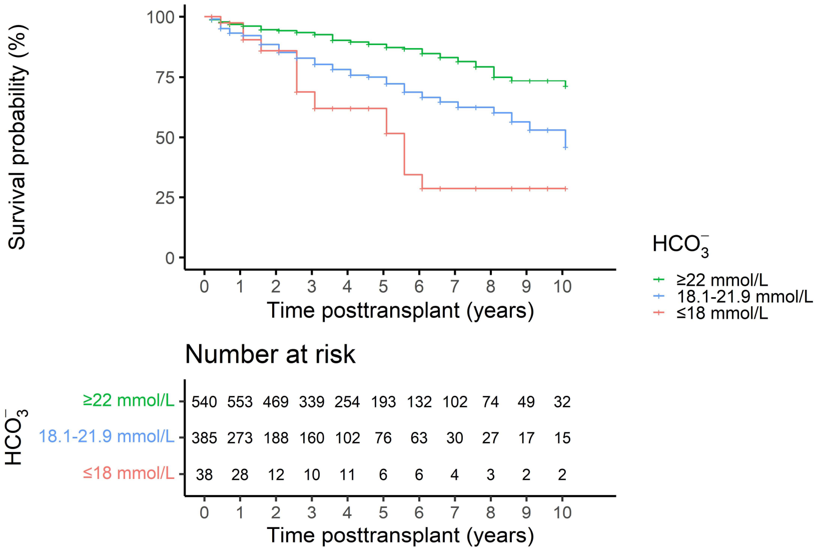

Reference: HCO_3^- ≥22 mmol/L

HCO_3^- 18.1- 21.9 mmol/L; HR, 2.28; 95% CI, 1.60 to 3.25; p<0.0001

HCO_3^- ≤18 mmol/L; HR, 4.51; 95% CI, 2.34 to 8.70; p<0.0001

Association between the cumulative incidence of time to composite endpoint and time-varying severe metabolic acidosis (red line), mild to moderate metabolic acidosis (blue line), and no acidosis (green line) in patients with normal anion gap

Number at risk corresponds to the number of patients with available HCO_3^- at a given time point.

CI, confidence interval; HR, hazard ratio

7. Figure S2

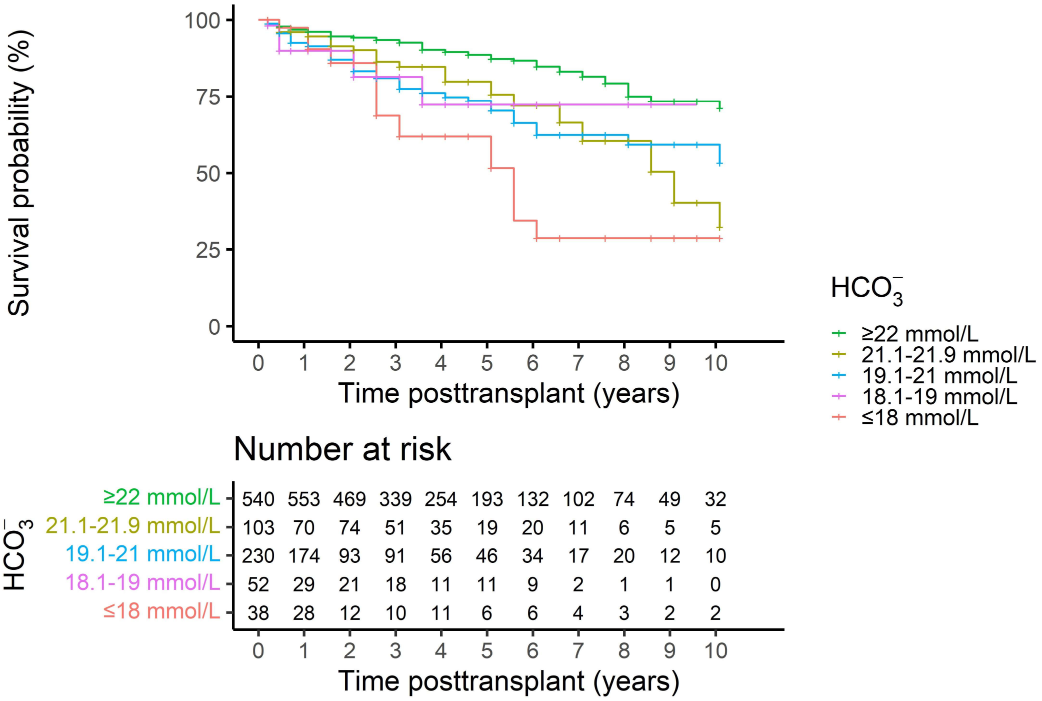

Reference: HCO_3^- ≥22 mmol/L

HCO_3^- 21.1- 21.9 mmol/L; HR, 1.93; 95% CI, 1.13 to 3.28; p=0.01

HCO_3^- 19.1- 21 mmol/L; HR, 2.40; 95% CI, 1.60 to 3.62; p<0.0001

HCO_3^- 18.1- 19 mmol/L; HR, 2.69; 95% CI, 1.28 to 5.66; p=0.008

HCO_3^- ≤18 mmol/L; HR, 4.58; 95% CI, 2.37 to 8.88; p<0.0001

Association between the degree of time-varying metabolic acidosis and time to composite endpoint in patients with normal anion gap

Number at risk corresponds to the number of patients with available HCO_3^- at a given time point.

CI, confidence interval; HR, hazard ratio

8. *Supplemental Table 1*

Analysis of factors associated with amelioration of metabolic acidosis under alkali supplementation at 3 months, 1, 3 and 5 years posttransplant (time since transplantation corresponds to the date of HCO_3^- measurement and the status of alkali supplementation at the previous time point).

	**Time since transplantation**							
	**3 months N= 295**		**1 year N= 362**		**3 years N= 265**		**5 years N= 171**	
Variable	Univariable OR (95% CI) p value	Multivariable OR (95% CI) p value	Univariable OR (95% CI) p value	Multivariable OR (95% CI) p value	Univariable OR (95% CI) p value	Multivariable OR (95% CI) p value	Univariable OR (95% CI) p value	Multivariable OR (95% CI) p value
Recipient age (years)	1.07 (1.02-1.12) p=0.003	1.05 (1.00-1.11) p=0.034	1.05 (1.01-1.09) p=0.03	1.07 (1.02-1.12) p=0.010	0.98 (0.93-1.03) p=0.45	1.0 (0.94-1.06) p=0.90	1.0 (0.94-1.06) p=0.99	0.98 (0.91-1.05) p=0.55
Female sex	0.80 (0.50-1.29) p=0.363	0.82 (0.50-1.34) p=0.43	0.93 (0.60-1.45) p=0.76	0.91 (0.56-1.50) p=0.72	1.14 (0.68-1.94) p=0.62	1.18 (0.64-2.21) p=0.59	0.76 (0.39-1.50) p=0.43	0.91 (0.44-1.92) p=0.80
Dose of alkali (mg/kg/day of sodium bicarbonate)	1.0 (0.99-1.00) p=0.02	1.0 (0.99-1.00) p=0.09	1.00 (1.00-1.00) p=0.82	1.00 (1.00-1.00) p=0.68	1.0 (1.00-1.00) p=0.42	1.0 (1.00-1.00) p=0.48	1.00 (1.00-1.01) p=0.43	1.00 (0.99-1.01) p=0.75
eGFR (mL/min per 1.73 m²)	0.99 (0.98-1.01) p=0.32	1.00 (0.99-1.01) p=0.98	1.00 (1.00-1.01) p=0.27	1.01 (1.00-1.02) p=0.14	1.01 (1.00-1.02) p=0.01	1.02 (1.01-1.03) p=0.003	1.00 (0.99-1.01) p=0.94	1.00 (0.99-1.02) p=0.84
